# 10KP: A phylodiverse genome sequencing plan

**DOI:** 10.1093/gigascience/giy013

**Published:** 2018-02-20

**Authors:** Shifeng Cheng, Michael Melkonian, Stephen A Smith, Samuel Brockington, John M Archibald, Pierre-Marc Delaux, Fay-Wei Li, Barbara Melkonian, Evgeny V Mavrodiev, Wenjing Sun, Yuan Fu, Huanming Yang, Douglas E Soltis, Sean W Graham, Pamela S Soltis, Xin Liu, Xun Xu, Gane Ka-Shu Wong

**Affiliations:** 1 BGI-Shenzhen, Shenzhen 518083, China; 2 China National GeneBank, BGI-Shenzhen, Shenzhen 518120, China; 3 Botanical Institute, Universität zu Köln, Cologne D-50674, Germany; 4 Department of Ecology and Evolutionary Biology, University of Michigan, Ann Arbor, MI 48109, USA; 5 Department of Plant Sciences, University of Cambridge, Tennis Court Road, Cambridge CB2 3EA, UK; 6 Centre for Comparative Genomics and Evolutionary Bioinformatics, Department of Biochemistry and Molecular Biology, Dalhousie University, Halifax NS, B3H 4R2 Canada; 7 Laboratoire de Recherche en Sciences Végétales, Université de Toulouse, UPS/CNRS, 24 chemin de Borde Rouge, Auzeville B.P. 42617, 31326 Castanet-Tolosan, France; 8 Boyce Thompson Institute, Ithaca, NY 14850, USA and Section of Plant Biology, Cornell University, Ithaca, NY 14853, USA; 9 Florida Museum of Natural History, University of Florida, PO Box 117800, Gainesville, FL 32611, USA; 10 James D. Watson Institute of Genome Sciences, Hangzhou 310058, China; 11 Department of Biology, University of Florida, Gainesville, FL 32611, USA; 12 Department of Botany, University of British Columbia, Vancouver BC, V6T 1Z4 Canada; 13 Department of Biological Sciences, University of Alberta, Edmonton AB, T6G 2E9 Canada; 14 Department of Medicine, University of Alberta, Edmonton AB, T6G 2E1 Canada

**Keywords:** 10KP, plants, samples, genome sequencing, genomics, biodiversity, phylogenomics, open community, MGISEQ

## Abstract

Understanding plant evolution and diversity in a phylogenomic context is an enormous challenge due, in part, to limited availability of genome-scale data across phylodiverse species. The 10KP (10,000 Plants) Genome Sequencing Project will sequence and characterize representative genomes from every major clade of embryophytes, green algae, and protists (excluding fungi) within the next 5 years. By implementing and continuously improving leading-edge sequencing technologies and bioinformatics tools, 10KP will catalogue the genome content of plant and protist diversity and make these data freely available as an enduring foundation for future scientific discoveries and applications. 10KP is structured as an international consortium, open to the global community, including botanical gardens, plant research institutes, universities, and private industry. Our immediate goal is to establish a policy framework for this endeavor, the principles of which are outlined here.

## Introduction

Based on the success of the 1KP (1000 Plants) Initiative [1,2], an international multidisciplinary consortium that sequenced and analyzed transcriptomes from more than 1000 species of green plants representing most of the known diversity within Viridiplantae, we are now aiming to sequence complete genomes from more than 10,000 plants and protists. 10KP will address fundamental questions in plant evolution and diversity, providing data on more than 10,000 species representing every major clade of embryophytes (land plants), green algae (chlorophytes and streptophytes), and protists (photosynthetic and heterotrophic). For embryophytes, we will sequence nonflowering plants (bryophytes, lycophytes, ferns, and gymnosperms) and flowering plants (angiosperms). In addition to green algae, we will also sequence diverse clades of photosynthetic and heterotrophic protists, representing some of the most enigmatic and unexplored eukaryotic microbes. This project was launched at the XIXth International Botanical Congress (2017, Shenzhen, China) and was covered by a *Science News* release in July 2017 [3].

Following the Bermuda Principles and the Fort Lauderdale Agreement, this project will make the resulting genomics data freely available. In the spirit of the Toronto Data Release Workshop and Statement [4] recommendation, which encourages large-scale sequencing projects to produce a citable statement for their data and intentions for downstream analyses and publications, we present this marker paper to outline our overall plans and explain how interested parties can get involved.

The basic goal of 10KP is to build an annotated reference genome for a member of every genus of the Viridiplantae (land plants and green algae), as well as a phylodiverse set of species representing both photosynthetic and heterotrophic protists. These data will provide a wealth of information to address fundamental questions across the plant/eukaryotic tree of life, e.g., enabling studies of phylogeny, origin/acquisition and diversification of specific traits, gene and genome duplication, correlation between genomic and morphological changes, and convergent evolution of important genetic networks. The scope and quantity of data produced by 10KP will allow researchers to develop new techniques that address fundamental questions in evolution and comparative genomics.

We will complete this project over the next 5 years (2018–2023), including sample acquisition, sequencing, genome assembly, analyses, and (initial) publications. Major supporters include BGI-Research, the nonprofit division of BGI-Shenzhen, and China National GeneBank (CNGB), an open nonprofit scientific platform that is managed by BGI-Shenzhen.

Through 10KP, we hope to foster imaginative and high-quality research that addresses major questions in plant and protist biology while also, indirectly, demonstrating the value of both preserving and investigating biodiversity. Our effort is meant to complement, not replace, research programs supported through other funding agencies worldwide. We are open to collaborations with all interested research groups.

## Species Lists and Phylogenetic Diversity

The number of extant species of Viridiplantae and protists is unknown. An estimate for embryophytes, gleaned from various databases (Table 1), is that there are at least 380,000 known species representing approximately 23,562 genera in 667 families; however, Govaerts [5] estimated a higher number. For green algae and photosynthetic protists, approximately 40,000 species have been described, and predictions for species yet to be described range from 25,000 to 100,000 [6]. In the case of heterotrophic eukaryotic microbes, estimates are much less clear; approximately 440,000 species have been described, most of which (approximately 400,000) are fungi [7]. The reality is that much of eukaryotic microbial diversity remains unexplored and the number of genera and species is essentially unknown.

**Table 1:** Statistics of the described species of embryophytes (land plants) distributed among major clades

Clade	Family	Genus	Species	Three largest families		
Hornworts	5	11	130	Anthocerotaceae (161)	Dendrocerotaceae (25)	Notothyladaceae (16)
Liverworts	87	387	7356	Lejeuneaceae (2270)	Jungermanniaceae(725)	Lepidoziaceae (580)
Mosses	111	874	13,000	Pottiaceae (3223)	Hypnaceae (2520)	Bryaceae (2108)
Lycophytes	3	18	1338	Lycopodiaceae (475)	Selaginellaceae (404)	Isoetaceae (51)
Ferns	48	319	10,578	Dryopteridaceae (1871)	Polypodiaceae (1601)	Pteridaceae (1226)
Gymnosperms	12	88	1104	Pinaceae (255)	Zamiaceae (216)	Cupressaceae (166)
ANA grade	6	20	193	Nymphaeaceae (88)	Schisandraceae (84)	Hydatellaceae (12)
Monocots	78	3505	76,119	Orchidaceae (28,576)	Poaceae (12,397)	Cyperaceae (6311)
Magnoliids	20	450	9528	Lauraceae (3106)	Piperaceae (2770)	Annonaceae (2174)
Asterids	143	9763	135,213	Asteraceae (38,700)	Rubiaceae (14,412)	Lamiaceae (8671)
Rosids	154	6582	101,245	Fabaceae (26,245)	Euphorbiaceae (6904)	Rosaceae (6626)
Basal eudicots	17	464	7535	Ranunculaceae (3119)	Proteaceae (1492)	Papaveraceae (1062)
Others	NA	NA	1579			
Embryophyta	667	23,562	381,425			

The numbers were combined and integrated from the Open Tree of Life and the Plant List (2013) [8], especially for flowering plants, with a particular focus on the “accepted species” for the nonflowering plants due to its classification difficulties. Some numbers are not the same or inconsistent across different databases. The aim of this table is simply to give a rough estimate of the species distribution in the major clades and the 3 largest families within each clade.

**Table 2:** Technologies/platforms for DNA library construction and sequencing

Platform/strategy	Insert size	Assemblers	Quality requirement for tissue samples	Minimum requirement for DNA quantity	Minimum requirement for DNA fragments	Assembly results
MGISEQ (hierarchical shotgun)	170/350 bp	SOAP *de novo*	Net weight of fresh plant tissue ≥4 g	2 μg	>20 k	construct contigs
	2–5 K			20 μg		construct scaffolds
	10 k			30 μg		
	20 k			50 μg		
	40 k			60 μg		
MGISEQ + 10X		Supernova2	50–75 mg dried or 100–200 mg fresh leaf tissue	2 ng	>50/100 k	construct scaffolds
MGISEQ + long fragment reads		To be developed	50–75 mg dried or 100–200 mg fresh leaf tissue	1 ng	>50/100 k	construct contigs
MGISEQ + 10X/long fragment reads + Hi-C			500 mg, living tissues/cells		>50/100 k	chromosome-scales

The MGISEQ platform, a BGI-developed next-generation sequencing technology based on DNA nanoballs and combinatorial probe anchor synthesis, will be combined with evolving methodologies for improving long-range contiguity, including linked-read technologies from 10X genomics or single-tube long fragment reads from Complete Genomics (a division of BGI). HiC libraries may also be used to construct chromosome-level genome reference assemblies for a member of every family or for other critical species selected by the consortium. Generally speaking, sample providers must prepare either (a) a minimum amount of DNA >50 μg and DNA fragments >20 kb or (b) a minimum amount of DNA >5 ng and DNA fragments >50 kb or preferably >100 kb. For (a), we will use the traditional hierarchical shotgun strategy by combining paired-end libraries with a series of mate-pair libraries. For (b), we will use the strategy of MGISEQ + 10X. The details of these platforms and the library/sequencing strategies are summarized in Table 2.

**Figure 1:**
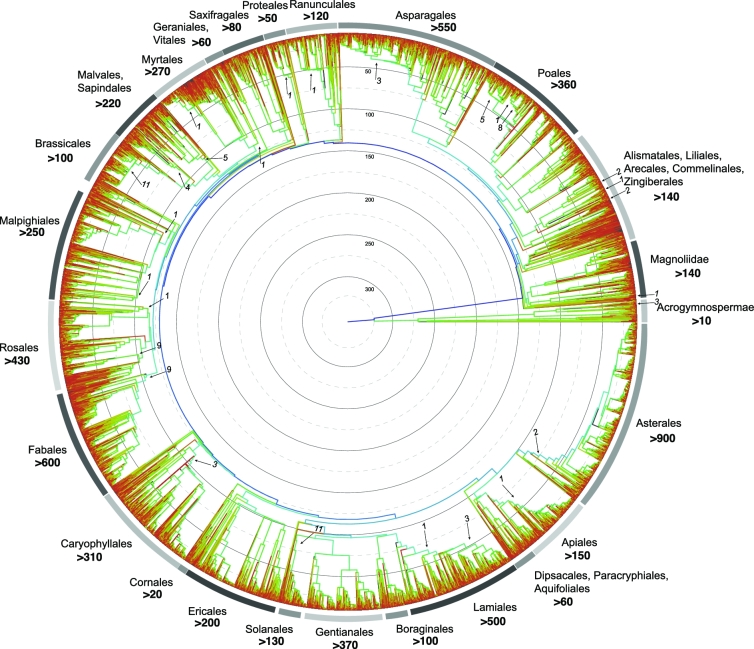
A phylogeny of seed plants (dated phylogeny based on Smith and Brown, in press). Colors correspond to the number of species in the subtending lineage (red = lower to blue = higher). Some larger clades are highlighted around the phylogeny along with the estimated number of genomes to be sequenced by 10KP in bold below the name. Smaller numbers and arrows inside the phylogeny indicate estimates of some of the already available genomes within the identified clade.

**Figure 2:**
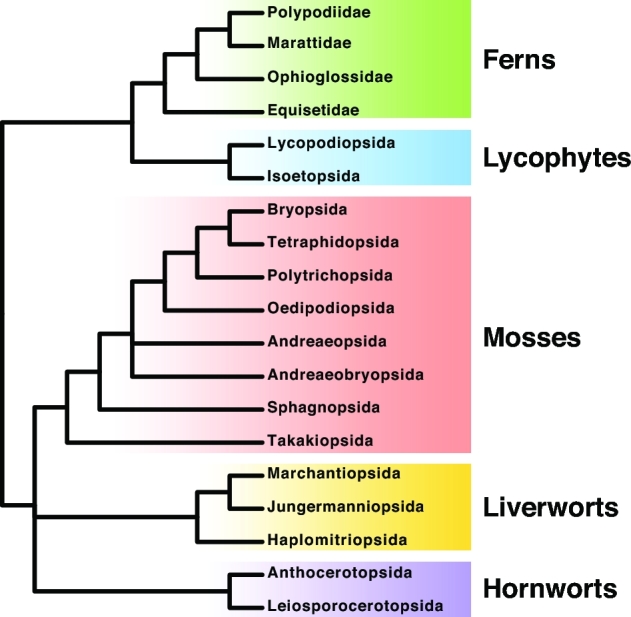
Summary tree of nonseed plants (based on the 1KP Capstone Analysis, in press). This shows the phylogenetic relationships for the 5 major categories of seed-free plants, including bryophytes (hornworts, liverworts, mosses), lycophytes, and ferns.

**Figure 3:**
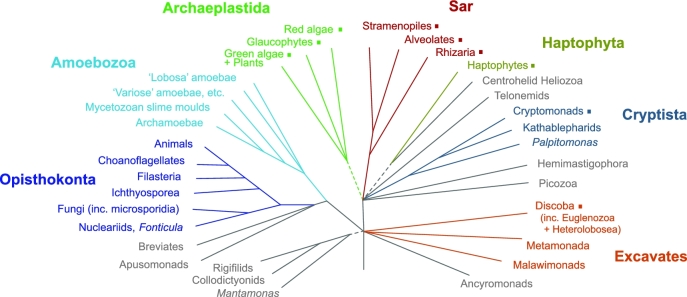
Summary tree of eukaryotes. Schematic diagram shows the known or predicted relationships among the major eukaryotic groups, based on multi-gene analyses, featuring diverse eukaryotic microbes (algae and protists) [10]. Lineages with 1 or more photosynthetic/plastid-bearing groups are highlighted with a square. The Archaeplastida are the eukaryotic “supergroup” to which green algae and embryophytes belong. Protist genomes sequenced as part of 10KP will come from diverse lineages but exclude true fungi and animals.

**Figure 4:**
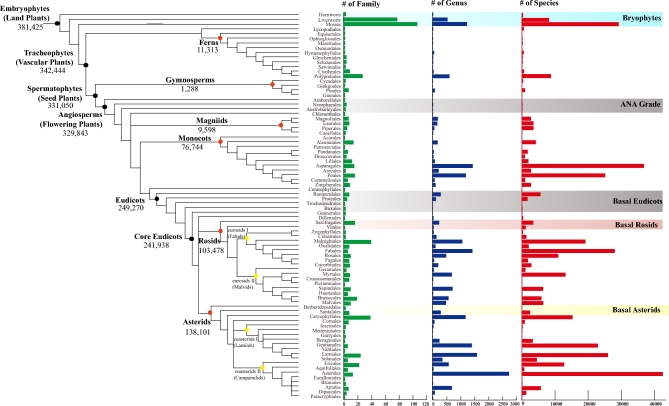
Distribution of species/genus/family abundance across the major clades of embryophytes. Most species belong to the eudicot clade, for which the largest families include Asteraceae, Orchidaceae, Fabaceae, Rubiaceae, and Poaceae.

**Figure 5:**
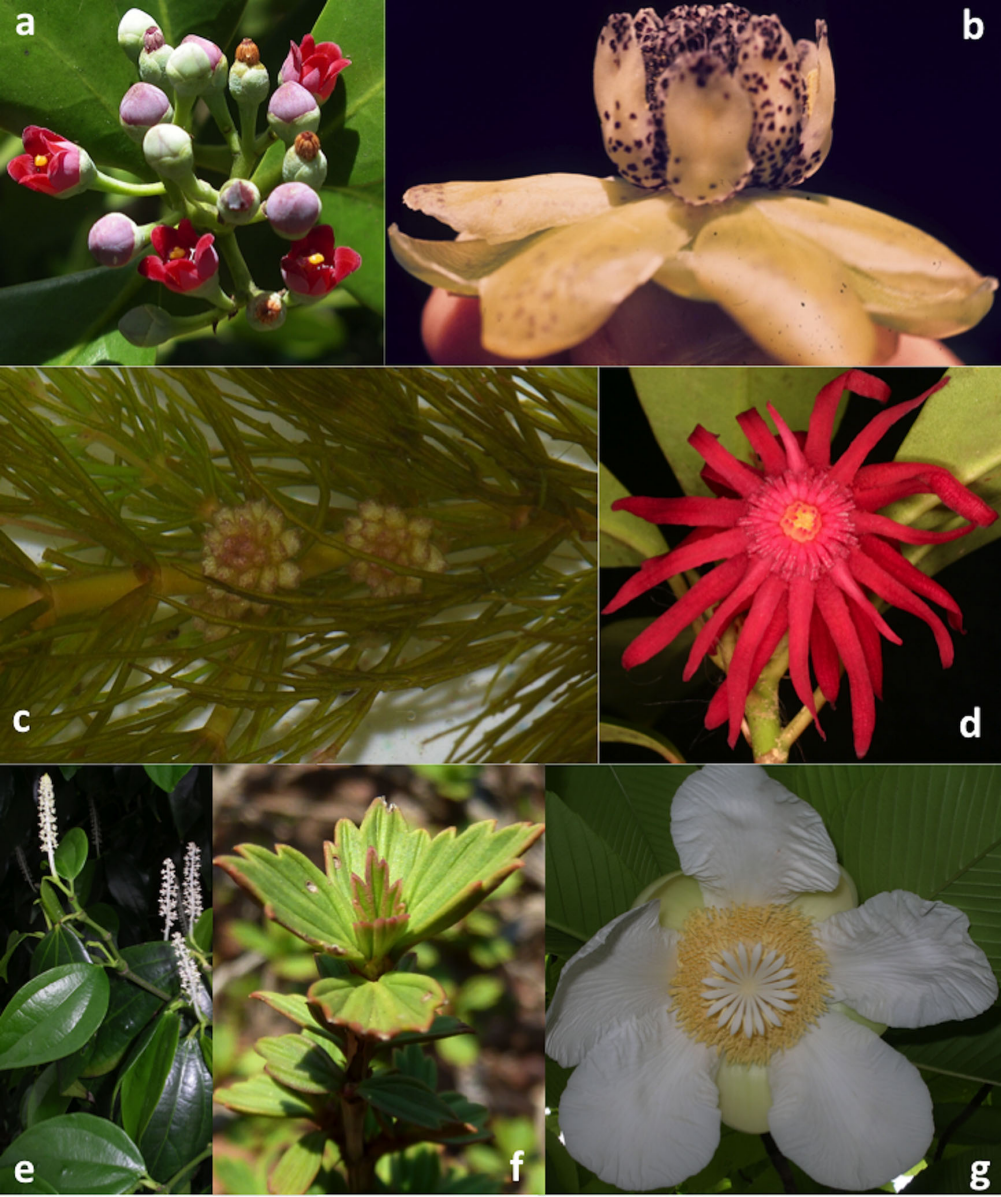
Representative images of species from different clades/families of flowering plants. The species names presented here are: a. *Canella winterana* (L.) Gaertn. (Angiosperms, Magnoliids, Canellales, Canellaceae). Flowers and inflorescence. Photo credit: Walter Judd. b. *Austrobaileya scandens* C.T. White (Angiosperms, Austrobaileyales, Austrobaileyaceae). Flower. Photo credit: Walter Judd. c. *Ceratophyllum demersum* L. (Angiosperms, Ceratophyllales, Ceratophyllaceae). Habit and inflorescence. Photo credit adapted from Christian Fischer, CC BY SA 3.0 Wikimedia Commons. d. *Illicium floridanum* J.Ellis (Angiosperms, Austrobaileyales, Schisandraceae). Flower. Photo credit: Walter Judd. e. *Piper neesianum* C. DC. (Angiosperms, Magnoliids, Piperales, Piperaceae). Habit and inflorescence. Photo credit: Walter Judd. f. *Myrothamnus flabellifolius* Welw. (Angiosperms, Eudicots, Gunnerales, Myrothamnaceae). Habit and leaves. Photo credit adapted from JMK, CC BY SA 3.0 Wikimedia Commons. g. *Dillenia indica* L. (Angiosperms, Eudicots, Dilleniales, Dilleniaceae). Photo credit: Walter Judd.

**Figure 6:**
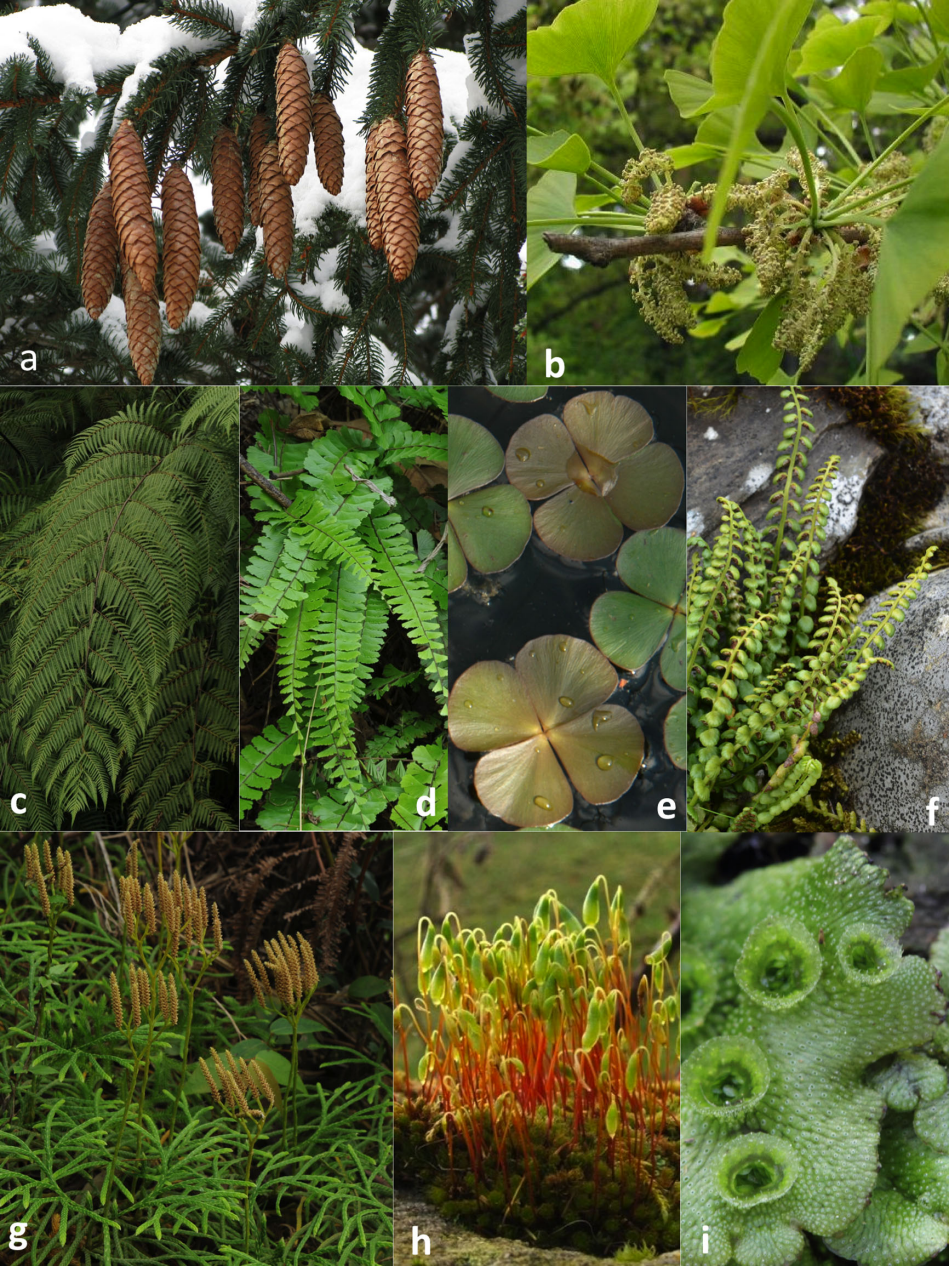
Representative images of species from various clades/families of nonflowering plants. The species names presented here are: a. *Picea abies* (L.) H. Karst. (Pinophyta, Pinales, Pinaceae). Shoots and female cones. Photo credit adapted from Magnus Manske (CC BY SA 3.0 Wikimedia Commons). b. *Ginkgo biloba* L. (Ginkgophyta, Ginkgoales, Ginkgoaceae). Leaves and male inflorescence. Photo credit adapted from Sten, CC-BY SA 3.0 Wikimedia Commons. c. *Cibotium barometz* (L.) J.Sm. (Polypodiopsida, Cyatheales, Cibotiaceae). Fronds (leaves). Photo credit: Pi-Fong Lu. d. *Adiantum caudatum* Klotzsch (Polypodiopsida, Polypodiales, Pteridaceae). Fronds (leaves) and habit. Photo credit: Pi-Fong Lu. e. *Marsilea crenata* C.Presl (Polypodiopsida, Salviniales, Marsileaceae). Fronds (leaves) and habit. Photo credit: Pi-Fong Lu. f. *Asplenium viride* Huds. (Polypodiopsida, Polypodiales, Aspleniaceae). Fronds (leaves) and habit. Photo credit: Pi-Fong Lu. g. *Diphasiastrum complanatum* (L.) Holub. (Lycopodiopsida, Lycopodiales, Lycopodiaceae. Habit. Photo credit: Pi-Fong Lu. h. *Bryum capilare* Hedwig (Bryopsida, Bryales, Bryaceae). Gametophyte and Sporophyte. Photo Credit adapted from Lairich Rig (CC BY SA 2.0 Wikimedia Commons). i. *Marchantia polymorpha* L. (Marchantiopsida, Marchantiales, Marchantiaceae). Thalli with gemmae (asexual reproductive structures). Photo Credit adapted from Holger Casselmann (CC-BY SA 3.0 Wikimedia Commons).

**Figure 7:**
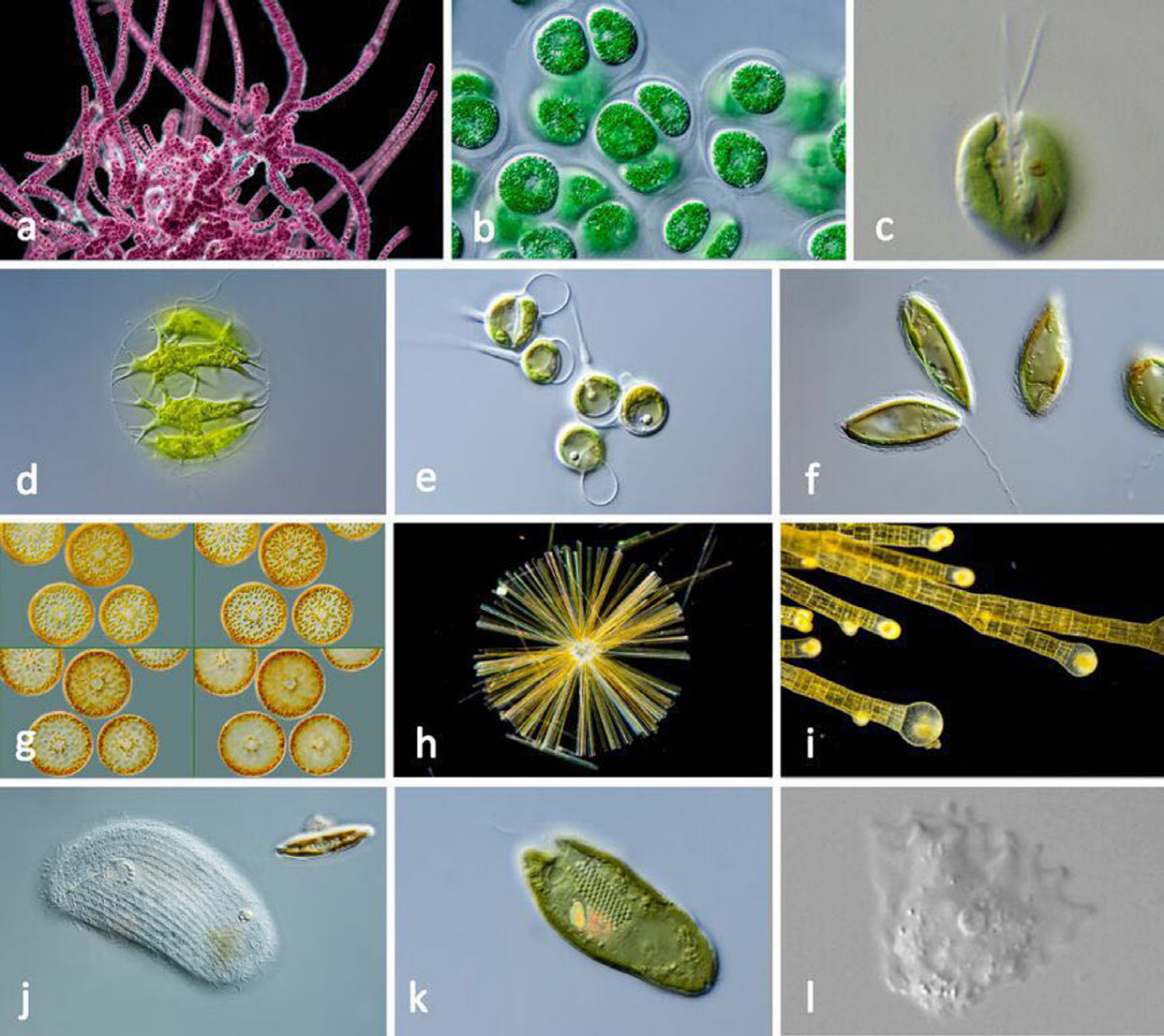
Light micrographs of diverse protists, including members of different eukaryotic “supergroups.” a. *Stylonema* (Archaeplastida [Plantae], red alga). b. *Cyanoptyche* (Archaeplastida [Plantae], glaucophyte). c. *Scherffelia* (Archaeplastida [Plantae], Viridiplantae, chlorophyte). d. *Stephanosphaera* (Archaeplastida [Plantae], Viridiplantae, chlorophyte). e. *Chaetosphaeridium* (Archaeplastida [Plantae], Viridiplantae, streptophyte). f. *Mallomonas* (stramenopiles, chrysophyte). g. *Coscinodiscus* (stramenopiles, diatom). h. *Synedra* (stramenopiles, diatom). i. *Sphacelaria* (stramenopiles, brown alga). j. *Trithigmostoma* (alveolates, ciliate). k. *Cryptomonas* (Cryptista). l. *Paramoeba* (Amoebozoa). Micrographs courtesy of Gerd Günther (http://www.mikroskopia.de/index.html), Sebastian Hess (Halifax; *Scherffelia*), and Ivan Fiala (Czech Republic; *Paramoeba*).

Fewer than 300 green plants and protists have had their genomes sequenced and published. The vast majority of the tree of life remains unexplored at the level of complete genomes. 10KP will fill this gap. Specifically, we will sequence genomes from at least 8000 seed-plant genera (Figure 1), at least 1000 nonseed plant genera (Figure 2), at least 1000 green algae, and at least 3000 photosynthetic and heterotrophic protists (Figure 3). The total number of species sequenced will exceed 10,000—the moniker 10KP is essentially a milestone toward a larger goal. We anticipate sequencing a proportionately larger number of eudicot genomes, given that this clade comprises nearly 75% of all angiosperm species (Figure 4). Many of the genomes to be sequenced are cornerstones for addressing important and longstanding questions in biology and evolution, while others represent unexplored potential for medicinal compounds and/or the discovery of high-value natural products. Some representative species from diverse clades are shown (Figures 5–7).

**Figure 8:**
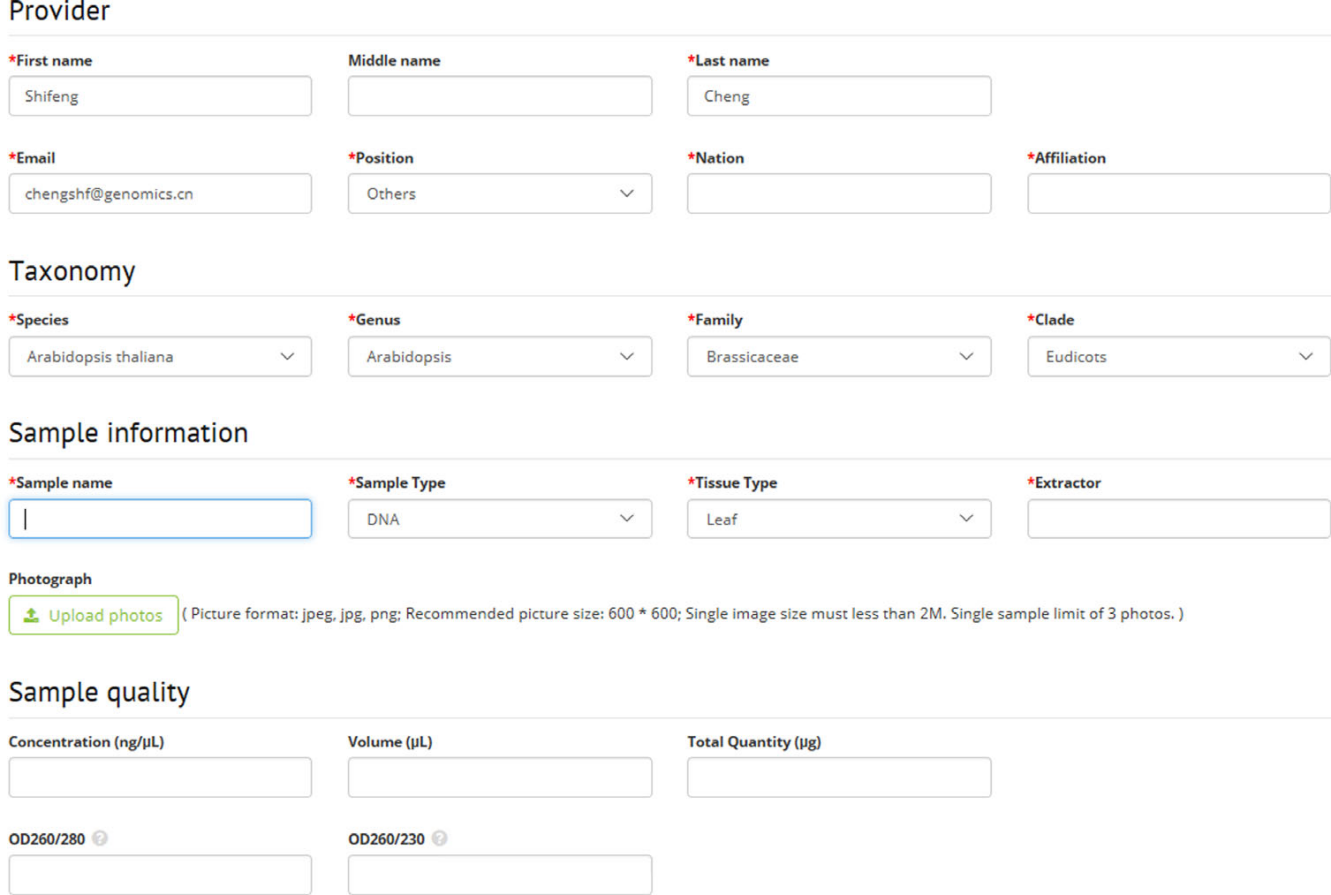
Sample submission portal from the CNGB/10KP website. Figure shows the sample submission portal (as well as the underlying database management) on the 10KP website https://db.cngb.org/10kp/; this website (version 1.0) is still evovling. This sample submission portal is prepared specifically for land plants; most samples will come from botanical gardens or botany research centers/laboratories worldwide. A global community effort is crucial to help supply all families and all genera. For stage 1, we anticipate more active involvement of highly motivated and skilled laboratories, whereas for stage 2, we anticipate more of a community effort to supply the majority of the remaining genera. For algae and protists, all samples will come from the public collections and channelled through the Culture Collection of Algae at the University of Cologne, where quality controls will be enforced.

## Sequencing Priority and Data Release

For embryophytes, we are expecting a community effort with sample submission and processing online (Figure 8). The species sampling will be coordinated by Douglas E. Soltis and Pamela S. Soltis (flowering plants) and Sean W. Graham (nonflowering plants). For this part, we will prioritize the sequencing in 2 stages:

*Stage 1.* Create family-level high-quality reference genomes, ideally with chromosome-scale assemblies to facilitate comparative and evolutionary genomics research across the green tree of life.
*Stage 2.* Increase the sample density to the genus level, while recognizing that many genera are likely not monophyletic. For some of the larger genera, we may sequence 2 or more distantly related species. Note that we will accept samples for genus-level sequencing even during the first phase of the project when we are focused on family-level sequencing, but these samples may be not processed immediately. We will, however, conduct appropriate quality controls before freezing the samples for later sequencing.

For green algae (chlorophytes and streptophytes) and photosynthetic protists, all samples will be channelled through public culture collections, specifically the Culture Collection of Algae at the University of Cologne (http://www.ccac.uni-koeln.de/) managed by Michael Melkonian and Barbara Melkonian, to ensure uniform quality control. For heterotrophic protists, project coordination will be led by researchers at the Centre for Comparative Genomics & Evolutionary Bioinformatics at Dalhousie University, Halifax, Canada (J. M. Archibald).

**Figure 9:**
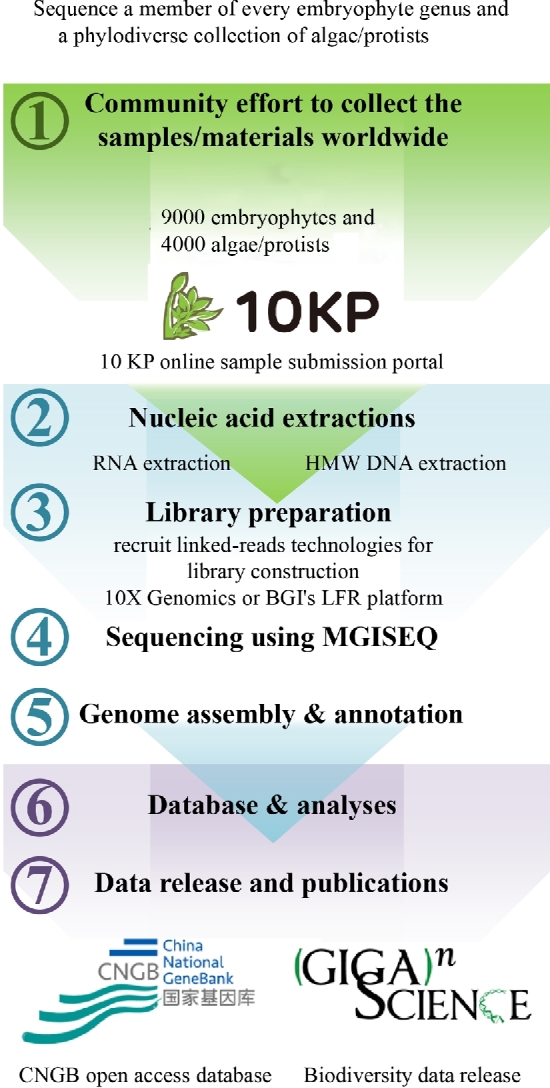
An overview of the 10KP strategy.

Annotated genome sequences will be released through the CNGB website (http://db.cngb.org/cnsa) and accompanied by regular submissions of peer-reviewed *GigaScience* “data release” publications that provide independent quality assessment and give credit/authorships to the appropriate sample providers. Data releases will occur on a regular basis (e.g., quarterly) once the data satisfy one of a series of tiered quality assessments (e.g., gene-sized contigs, sufficient for synteny analysis, chromosome-scale assembly). Additional increases in sample density may be coordinated with the larger Earth Biogenome Project [9], which will likely encompass at least another half decade of effort. A brief workflow for the 10KP is described (Figure 9).

## Sample Requirements and Sequencing Technologies

It is essential that proper sample documentation be provided. This includes vouchers for taxonomic verification (embryophytes and eukaryotic microbes are dealt with differently), provenance data (detailing the source, origin, and geo-location of the species for sequencing), and prior informed consent for genome sequencing and data release (from appropriate authorities in compliance with the Nagoya protocol). For green algae and photosynthetic protists, the biological specimen is the strain (with unique numerical identifier) kept in a public repository (culture collection); no strain will be sequenced in 10KP that has not previously been deposited in a public culture collection. For heterotrophic protists, the situation is more difficult as many are difficult to grow and protist collections often lack the resources to keep these difficult strains in a living state. Good communication within the 10KP consortium and with external partners will be critical.

All of the sequencing will be conducted on BGI’s low-cost high-accuracy high-throughput MGISEQ platform, in combination with newly developed linked-read technologies (e.g., 10X genomics or BGI’s single-tube long fragment reads phasing technology). These approaches require much smaller amounts of DNA (only 2 ng/library) than traditional methods but they also require high-quality high-molecular-weight (>50 kb) extractions. The dominant alternative is the traditional “hierarchical shotgun” strategy with a series of mate-pair large-insert libraries. However, this approach requires a much larger amount of DNA (e.g., >100 μg), which is prohibitive for many plant species. We note that while our stated goal is to build high-quality reference genomes, transcriptome data are important for genome annotation; if live tissues are available, we will also sequence transcriptomes.

## Collaborative Proposals

10KP will also consider collaborative proposals that build on the existing dataset and generate new data using the MGISEQ platform. The aim of these projects should be to go beyond covering the diversity of species and, instead, to address important questions in basic and applied science. Up to 20% of the 10KP sequencing capacity will be devoted to these collaborations. Potential collaborators who wish to launch major subprojects within 10KP should provide a brief (maximum 5 pages) proposal as examplified in the supplementary template (proposal template for subprojects included as supplementary file).

## Additional file

Supplementary file, proposal template.docx.

## Abbreviations

10KP: 10,000 Plants Genome Sequencing Project; CNGB: China National Gene Bank.

## Competing interests

S.C., W.S., Y.F., H.Y., X.L., and X.X. are employees of BGI Shenzhen. The authors otherwise declare that they have no competing interests.

## Supplementary Material

GIGA-D-18-00055_Original_Submission.pdf
