# Supplementary material for: 10KP: A phylodiverse genome sequencing plan
Source: Gigascience. 2018 Feb 20;7(3):giy013. doi: 10.1093/gigascience/giy013 (PMC5869286; doi:10.1093/gigascience/giy013)
Supplement: GIGA-D-18-00055_Original_Submission.pdf [file giy013_giga-d-18-00055_original_submission.pdf]

GigaScience

10KP: A Phylodiverse Genome Sequencing Plan

--Manuscript Draft--

**Manuscript Number:**	GIGA-D-18-00055
**Full Title:**	10KP: A Phylodiverse Genome Sequencing Plan
**Article Type:**	Commentary
**Funding Information:**	Shenzhen Municipal Government of China (JCYJ20150529150409546) — Dr. Shifeng Cheng<br>Shenzhen Municipal Government of China (JCYJ20150529150505656) — Dr. Xin Liu
**Abstract:**	Understanding plant evolution and diversity in a phylogenomic context is an enormous challenge, due in part to limited availability of genome-scale data across phylodiverse species. The 10KP (10,000 Plants) Genome Sequencing Project will sequence and characterize representative genomes from every major clade of embryophytes, green algae, and protists (excluding fungi) within the next five years. By implementing (and continuously improving) leading-edge sequencing technologies and bioinformatics tools, 10KP will catalogue the genome content of plant and protist diversity, and make these data freely available as an enduring foundation for future scientific discovery and application. 10KP is structured as an international consortium, open to the global community, including botanical gardens, plant research institutes, universities, and private industry. Our immediate goal is to establish a policy framework for this endeavour, the principles of which are outlined here.
**Corresponding Author:**	Shifeng Cheng<br><br>CHINA
**Corresponding Author Secondary Information:**	
**Corresponding Author's Institution:**	
**Corresponding Author's Secondary Institution:**	
**First Author:**	Shifeng Cheng
**First Author Secondary Information:**	
**Order of Authors:**	Shifeng Cheng
	Michael Melkonian
	Stephen Smith
	Samuel Brockington
	John M. Archibald
	Pierre-Marc Delaux
	Fay-wei Li
	Barbara Melkonian
	Evgeny V. Mavrodiev
	Wenjing Sun
	Yuan Fu
	Huanming Yang
	Douglas E. Soltis
	Sean W. Graham

Powered by Editorial Manager® and ProduXion Manager® from Aries Systems Corporation

	Pamela S. Soltis
	Xin Liu
	Xun Xu
	Gane Ka-Shu Wong
**Order of Authors Secondary Information:**	
**Opposed Reviewers:**	
**Additional Information:**	
**Question**	**Response**
Are you submitting this manuscript to a special series or article collection?	No
**Experimental design and statistics**<br><br>Full details of the experimental design and statistical methods used should be given in the Methods section, as detailed in our Minimum Standards Reporting Checklist. Information essential to interpreting the data presented should be made available in the figure legends.<br><br>Have you included all the information requested in your manuscript?	No
If not, please give reasons for any omissions below.<br><br>as follow-up to "**Experimental design and statistics**<br><br>Full details of the experimental design and statistical methods used should be given in the Methods section, as detailed in our Minimum Standards Reporting Checklist. Information essential to interpreting the data presented should be made available in the figure legends.<br><br>Have you included all the information requested in your manuscript?<br><br>"	This is a commentary manuscript
**Resources**<br><br>A description of all resources used, including antibodies, cell lines, animals and software tools, with enough information to allow them to be uniquely identified, should be included in the Methods section. Authors are strongly encouraged to cite Research Resource	No

Identifiers (RRIDs) for antibodies, model organisms and tools, where possible.<br><br>Have you included the information requested as detailed in our Minimum Standards Reporting Checklist?	
If not, please give reasons for any omissions below.<br><br>as follow-up to "**Resources**<br><br>A description of all resources used, including antibodies, cell lines, animals and software tools, with enough information to allow them to be uniquely identified, should be included in the Methods section. Authors are strongly encouraged to cite Research Resource Identifiers (RRIDs) for antibodies, model organisms and tools, where possible.<br><br>Have you included the information requested as detailed in our Minimum Standards Reporting Checklist?<br><br>"	This is a commentary manuscript
**Availability of data and materials**<br><br>All datasets and code on which the conclusions of the paper rely must be either included in your submission or deposited in publicly available repositories (where available and ethically appropriate), referencing such data using a unique identifier in the references and in the "Availability of Data and Materials" section of your manuscript.<br><br>Have you have met the above requirement as detailed in our Minimum Standards Reporting Checklist?	No
If not, please give reasons for any omissions below.<br><br>as follow-up to "**Availability of data and materials**<br><br>All datasets and code on which the conclusions of the paper rely must be either included in your submission or	This is a commentary manuscript

deposited in publicly available repositories (where available and ethically appropriate), referencing such data using a unique identifier in the references and in the “Availability of Data and Materials” section of your manuscript.<br><br>Have you have met the above requirement as detailed in our Minimum Standards Reporting Checklist?<br><br>"	

Manuscript
Click here to download Manuscript 10KP, policy manuscript, main text.doc

10KP: A Phylodiverse Genome Sequencing Plan

Shifeng Cheng[1,2*], Michael Melkonian[3], Stephen A. Smith[4], Samuel Brockington[5], John M. Archibald[6], Pierre-Marc Delaux[7], Fay-wei Li[8], Barbara Melkonian[3], Evgeny V. Mavrodiev[9], Wenjing Sun[1,2], Yuan Fu[1,2], Huanming Yang[1,10], Douglas E. Soltis[9,11], Sean W. Graham[12], Pamela S. Soltis[9,11], Xin Liu[1,2*], Xun Xu[1,2#], Gane Ka-Shu Wong[1,13,14#]

[1]BGI-Shenzhen, Shenzhen 518083, China

[2]China National GeneBank, BGI-Shenzhen, Shenzhen, 518120, China.

[3]Botanical Institute, Universität zu Köln, Cologne D-50674, Germany

[4]Department of Ecology and Evolutionary Biology, University of Michigan, Ann Arbor, Michigan 48109, USA

[5]Department of Plant Sciences, University of Cambridge, Tennis Court Road, Cambridge CB2 3EA, UK.

[6]Centre for Comparative Genomics and Evolutionary Bioinformatics, Department of Biochemistry and Molecular Biology, Dalhousie University, Halifax, NS B3H 4R2, Canada

[7]Laboratoire de Recherche en Sciences Végétales, Université de Toulouse, CNRS, UPS, 24 chemin de Borde Rouge, Auzeville, BP42617, 31326 Castanet Tolosan, France

[8]Boyce Thompson Institute, Ithaca, NY 14850, USA & Section of Plant Biology, Cornell University

[9]Florida Museum of Natural History, University of Florida, PO Box 117800, Gainesville, FL 32611, USA.

[10]James D. Watson Institute of Genome Sciences, Hangzhou 310058, China

[11]Department of Biology, University of Florida, Gainesville, FL, 32611 USA

[12]Department of Botany, University of British Columbia, Vancouver BC, V6T 1Z4 Canada

[13]Department of Biological Sciences, University of Alberta, Edmonton AB, T6G 2E9 Canada

[14]Department of Medicine, University of Alberta, Edmonton AB, T6G 2E1 Canada;

*These authors contributed equally to this work

[#]To whom correspondence should be addressed: Emails: gane@ualberta.ca and xuxun@genomics.cn

Abstract

Understanding plant evolution and diversity in a phylogenomic context is an enormous challenge, due in part to limited availability of genome-scale data across phylodiverse species. The 10KP (10,000 Plants) Genome Sequencing Project will sequence and characterize representative genomes from every major clade of embryophytes, green algae, and protists (excluding fungi) within the next five years. By implementing (and continuously improving) leading-edge sequencing technologies and bioinformatics tools, 10KP will catalogue the genome content of plant and protist diversity, and make these data freely available as an enduring foundation for future scientific discovery and application. 10KP is structured as an international consortium, open to the global community, including botanical gardens, plant research institutes, universities, and private industry. Our immediate goal is to establish a policy framework for this endeavour, the principles of which are outlined here.

Keywords: 10KP, Plants, Samples, Genome Sequencing, Genomics, Biodiversity, Phylogenomics, Open Community, MGISEQ

Introduction

Based on the success of the 1KP project[1,2], an international multi-disciplinary consortium that sequenced and analysed transcriptomes from over 1,000 species of green plants representing most of the known diversity within Viridiplantae, we are now aiming to sequence complete genomes from over 10,000 plants and protists. 10KP will address fundamental questions in plant evolution and diversity, providing data on over 10,000 species representing every major clade of embryophytes (land plants), green algae (chlorophytes and streptophytes), and protists (photosynthetic and heterotrophic). For embryophytes, we will sequence non-flowering plants (bryophytes, lycophytes, ferns and gymnosperms) and flowering plants (angiosperms). In addition to green algae, we will also sequence diverse clades of photosynthetic and heterotrophic protists, representing some of the most enigmatic and unexplored eukaryotic microbes. The project was launched at the XIXth International Botanical Congress (IBC2017, Shenzhen) and was covered by a Science News release in July of 2017[3].

Following the Bermuda Principles and the Fort Lauderdale agreement, this project will make the resulting genomics data freely available. In the spirit of the Toronto Data Release Workshop and Statement[4] recommendation, which encourages large-scale sequencing projects to produce a citable statement for their data and intentions for downstream analyses and publications, we present this marker paper to outline our overall plans and explain how interested parties can get involved.

The basic goal of 10KP is to build an annotated reference genome for a member of every genus of the Viridiplantae (land plants and green algae), as well as a phylodiverse set of species representing both photosynthetic and heterotrophic protists. These data will provide a wealth of information to address fundamental questions across the plant/eukaryotic tree of life, e.g., enabling studies of phylogeny, origin/acquisition and diversification of specific traits, gene and genome duplication, correlation between genomic and morphological changes, and convergent evolution of important genetic networks. The scope and quantity of data produced by 10KP will allow researchers to develop new techniques that address fundamental questions in evolution and comparative genomics.

We will complete this project over the next five years (2018-2023), including sample acquisition, sequencing, genome assembly, analyses and (initial) publications. Major supporters

include BGI-Research, the non-profit division of BGI-Shenzhen, and China National GeneBank (CNGB), an open non-profit scientific platform that is managed by BGI-Shenzhen.

Through 10KP, we hope to foster imaginative and high-quality research that addresses major questions in plant and protist biology while also, indirectly, demonstrating the value of both preserving and investigating biodiversity. Our effort is meant to complement, not replace, research programs supported through other funding agencies worldwide. We are open to collaborations with all interested research groups.

Species Lists and Phylogenetic Diversity

The number of extant species of Viridiplantae and protists is unknown. An estimate for embryophytes, gleaned from various databases (**Table 1**), is that there are at least 380,000 known species representing approximately 23,562 genera in 667 families; however, Govaerts[5] estimated a higher number. For green algae and photosynthetic protists, approximately 40,000 species have been described, and predictions for species yet to be described range from 25,000 to 100,000[6]. In the case of heterotrophic eukaryotic microbes, estimates are much less clear; approximately 440,000 species have been described, most of which (~400,000) are fungi[7]. The reality is that much of eukaryotic microbial diversity remains unexplored and the number of genera and species is essentially unknown.

Fewer than 300 green plants and protists have had their genomes sequenced and published. The vast majority of the tree of life remains unexplored at the level of complete genomes. 10KP will fill this gap. Specifically, we will sequence genomes from at least 8,000 seed-plant genera (**Figure 1**), at least 1,000 non-seed plant genera (**Figure 2**), at least 1,000 green algae, and at least 3,000 photosynthetic and heterotrophic protists (**Figure 3**). The total number of species sequenced will exceed 10,000 – the moniker 10KP is essentially a milestone towards a larger goal. We anticipate sequencing a proportionately large number of eudicot genomes, given that this clade comprises nearly 75% of all angiosperm species (**Figure 4**). Many of the genomes to be sequenced are cornerstones for addressing important and longstanding questions in biology and evolution, while others represent unexplored potential for medicinal compounds and/or the discovery of high-value natural products. Some representative species from diverse clades are shown (**Figure 5-7**).

Sequencing Priority and Data Release

For embryophytes, we are expecting a community effort with sample submission and processing online (**Figure 8**). The species sampling will be coordinated by Douglas E. Soltis and Pamela S. Soltis (flowering plants) and Sean W. Graham (non-flowering plants). For this part, we will prioritize the sequencing in two stages:

Stage 1. Create family-level high-quality reference genomes, ideally with chromosome-scale assemblies to facilitate comparative and evolutionary genomics research across the green tree-of-life.

Stage 2. Increase the sample density to the genus level, while recognizing that many genera are likely not monophyletic. For some of the larger genera, we may sequence two or more distantly-related species. Note that we will accept samples for genus-level sequencing even during the first phase of the project when we are focused on family-level sequencing, but these samples may be not processed immediately. We will however conduct appropriate quality controls before freezing the samples for later sequencing.

For green algae (chlorophytes and streptophytes) and photosynthetic protists, all samples will be channelled through public culture collections, specifically the CCAC (http://www.ccac.uni-koeln.de/) managed by Michael Melkonian and Barbara Melkonian, to ensure uniform quality control. For heterotrophic protists, project coordination will be led by researchers at the Centre for Comparative Genomics & Evolutionary Bioinformatics at Dalhousie University, Halifax, Canada (J. M. Archibald).

Annotated genome sequences will be released through the CNGB website (http://db.cngb.org/cnsa), and accompanied by regular submissions of peer-reviewed *GigaScience* “data release” publications that provide independent quality assessment and give credit/authorships to the appropriate sample providers. Data releases will occur on a regular basis (e.g. quarterly) once the data satisfy one of a series of tiered quality assessments (e.g. gene-sized contigs, sufficient for synteny analysis, chromosome-scale assembly). Additional increases in sample density may be coordinated with the larger Earth Biogenome Project[10], which will likely encompass at least another half decade of effort. A brief workflow for the 10KP is described (**Figure 9**).

Sample Requirements and Sequencing Technologies

It is essential that proper sample documentation be provided. This includes vouchers for taxonomic verification (embryophytes and eukaryotic microbes are dealt with differently), provenance data (detailing the source, origin, and geo-location of the species for sequencing), and prior informed consent for genome sequencing and data release (from appropriate authorities in compliance with the Nagoya protocol). For green algae and photosynthetic protists, the biological specimen is the strain (with unique numerical identifier) kept in a public repository (culture collection); no strain will be sequenced in 10KP that has not previously been deposited in a public culture collection. For heterotrophic protists, the situation is more difficult as many

are difficult to grow and protist collections often lack the resources to keep these difficult strains in a living state. Good communication within the 10KP consortium and with external partners will be critical.

All of the sequencing will be conducted on BGI's low-cost, high-accuracy and high-throughput MGISEQ platform, in combination with newly developed linked-read technologies (e.g., 10X genomics or BGI's single-tube Long Fragment Read phasing technology- stLFR). These approaches require much smaller amounts of DNA (only 2 ng/library) than traditional methods, but they also require high-quality high-molecular-weight (HMW; >50 kb) extractions. The dominant alternative is the traditional 'hierarchical shotgun' strategy with a series of mate-pair large-insert libraries. However, this approach requires a much larger amount of DNA (e.g., >100 ug), which is prohibitive for many plant species. We note that while our stated goal is to build high-quality reference genomes, transcriptome data are important for genome annotation, and if live tissues are available, we will also sequence transcriptomes.

Collaborative Proposals

10KP will consider collaborative proposals that build on the existing data set and generate new data using the MGISEQ platform. The aim of these projects should be to go beyond covering the diversity of species and, instead, to address important questions in basic and applied science. Up to 20% of the 10KP sequencing capacity will be devoted to these collaborations. Potential collaborators who wish to launch major subprojects within 10KP should provide a brief (maximum 5 pages) proposal as examplified in the supplementary template (Proposal template for subprojects included as supplementary file).

Competing Interests

S.C., W.S., Y.F., H.Y., X.L., X.X., and G.K.S.W. are employees of BGI Shenzhen. The authors otherwise declare that they have no competing interests.

Acknowledgements

This manuscript was mostly written by Shifeng Cheng and Gane Ka-Shu Wong, with extensive input and polishing from all of the authors listed in this consortium. We acknowledge many individuals who provided support in various aspects. Min Liu, Fengzhen Chen, Lei Chen, Xiaofeng Wei and Sanjie Jiang from BGI contributed to the construction of the 10KP website.

This project was supported by the Shenzhen Municipal Government of China (NO. JCYJ20150529150409546, NO. JCYJ20150529150505656).

Abbreviations

1KP: 1,000 Plant Project (transcriptomes); 10KP: 10,000 Plant Genome Sequencing project; CCAC: Culture Collection of Algae at the University of Cologne; CNGB: China National Gene Bank; cPAS: combinatorial Probe Anchor Synthesis; HMW: High Molecular Weight; IBC: International Botanical Congress; LFR: Long Fragment Reads; NGS: Next-Generation Sequencing technology; stLFR: single tube Long Fragment Reads.

References

1 1000 plant transcriptome consortium (1KP). www.onekp.com/

2 Norman J Wickett, et al. and Gane KS Wong, Jim Leebens-Mack. Nov 2014. Phylotranscriptomic analysis of the origin and early diversification of land plants. Proc. Natl. Acad. Sci. USA 111: E4859-E4868. PMID: 25355905

3 Normile, D. Plant scientists plan massive effort to sequence 10,000 genomes, <http://www.sciencemag.org/news/2017/07/plant-scientists-plan-massive-effort-sequence-10000-genomes> (2017).

4 Prepublication data sharing. Toronto International Data Release Workshop Authors. doi:10.1038/461168a

5 Govaerts, R. How many species of seed plants are there?-a response. *Taxon* **52**, 583-584 (2003).

6 Guiry, M. D. How many species of algae are there? *Journal of Phycology* **48**, 1057-1063 (2012).

7 Sina M. Adl, Brian S. Leander, Alastair G. B. Simpson, John M. Archibald et al. Diversity, Nomenclature, and Taxonomy of Protists. Syst. Biol. 56: 684-689 (2007)

8 The Plant List, a working list of all plant species. http://www.theplantlist.org/

9 Simpson, A., Slamovits, C. H. & Archibald, J. M. Protist Diversity and Eukaryote Phylogeny. (2017).

10 Pennisi, E. Biologists propose to sequence the DNA of all life on Earth, <http://www.sciencemag.org/news/2017/02/biologists-propose-sequence-dna-all-life-earth> (2017).

Supplementary Figures

Figure 1. A phylogeny of seed plants (dated phylogeny based on Smith and Brown, in press). Colours correspond to the number of species in the subtending lineage (red=lower to

blue=higher). Some larger clades are highlighted around the phylogeny along with the estimated number of genomes to be sequenced in 10KP in bold below the name. Smaller numbers and arrows inside the phylogeny indicate estimates of some of the already available genomes within the identified clade.

Figure 2. **Summary tree of non-seed plants** (based on 1KP capstone analysis, in press). Summary of phylogenetic relationships for the five major categories of seed-free plants, including bryophytes (hornworts, liverworts, mosses), lycophytes, and ferns.

Figure 3. **Summary tree of eukaryotes**. Schematic diagram shows the known or predicted relationships among the major eukaryotic groups, based on multi-gene analyses, featuring diverse eukaryotic microbes (algae and protists)[9]. Lineages with one or more photosynthetic/plastid-bearing groups are highlighted with a square. The Archaeplastida are the eukaryotic 'supergroup' to which green algae and embryophytes belong. Protist genomes sequenced as part of 10KP will come from diverse lineages, but exclude true fungi and animals.

Figure 4. Distribution of species/genus/family abundance across the major clades of embryophytes. Most species represent the eudicot clade, in which the largest families include Asteraceae, Orchidaceae, Fabaceae, Rubiaceae, and Poaceae.

Figure 5. Representative images of species from different clades/families of flowering plants. The species names presented here are: a. *Canella winterana* (L.) Gaertn. (Angiosperms, Magnoliids, Canellales, Canellaceae). Flowers and inflorescence. Photo Credit: Walter Judd; b. *Austrobaileya scandens* C.T.White (Angiosperms, Austrobaileyales, Austrobaileyaceae). Flower. Photo credit: Walter Judd; c. *Ceratophyllum demersum* L. (Angiosperms, Ceratophyllales, Ceratophyllaceae). Habit and inflorescence. Photo Credit adapted from Christian Fischer, CC BY SA 3.0 Wikimedia Commons ; d. *Illicium floridanum* J.Ellis (Angiosperms, Austrobaileyales, Schisandraceae). Flower. Photo credit: Walter Judd; e. *Piper neesianum* C. DC. (Angiosperms, Magnoliids, Piperales, Piperaceae). Habit and inflorescence. Photo credit: Walter Judd; f. *Myrothamnus flabellifolius* Welw. (Angiosperms, Eudicots, Gunnerales, Myrothamnaceae). Habit and leaves. Photo Credit adapted from Photo Credit adapted from JMK, CC BY SA 3.0 Wikimedia Commons; g. *Dillenia indica* L. (Angiosperms, Eudicots, Dilleniales, Dilleniaceae). Photo credit: Walter Judd.

Figure 6. Representative images of species from various clades/families of non-flowering plants. The species names presented here are: a. *Picea abies* (L.) H. Karst. (Pinophyta, Pinales,

Pinaceae). Photo Credit adapted from Magnus Manske, (CC BY SA 3.0 Wikimedia Commons) Shoots and female cones; b. *Ginkgo biloba* L. (Ginkgophyta, Ginkgoales, Ginkgoaceae). Leaves and male inflorescence. Photo credit adapted from Sten, CC-BY SA 3.0 Wikimedia Commons; c. *Cibotium barometz* (L.) J.Sm. (Polypodiopsida, Cyatheales, Cibotiaceae). Fronds (leaves). Photo Credit: Pi-Fong Lu; d. *Adiantum caudatum* Klotzsch (Polypodiopsida, Polypodiales, Pteridaceae). Fronds (leaves) and habit. Photo credit: Pi-Fong Lu; e. *Marsilea crenata* C.Presl (Polypodiopsida, Salviniales, Marsileaceae). Fronds (leaves) and habit. Photo credit: Pi-Fong Lu; f. *Asplenium viride* Huds. (Polypodiopsida, Polypodiales, Aspleniaceae). Fronds (leaves) and habit. Photo credit: Pi-Fong Lu; g. *Diphasiastrum complanatum* (L.) Holub. (Lycopodiopsida, Lycopodiales, Lycopodiaceae. Habit. Photo credit: Pi-Fong Lu; h. *Bryum capilare* Hedwig (Bryopsida, Bryales, Bryaceae). Gametophyte and Sporophyte. Photo Credit adapted from Lairich Rig (CC BY SA 2.0 Wikimedia Commons) ; i. *Marchantia polymorpha* L. (Marchantiopsida, Marchantiales, Marchantiaceae). Thalli with gemmae (asexual reproductive structures). Photo Credit adapted from Holger Casselmann (CC-BY SA 3.0 Wikimedia Commons).

Figure 7. Light micrographs of diverse protists, including members of different eukaryotic "supergroups": (a) *Stylonema* (Archaeplastida [Plantae], red alga), (b) *Cyanoptyche* (Archaeplastida [Plantae], glaucophyte), (c) *Scherffelia* (Archaeplastida [Plantae],Viridiplantae, chlorophyte), (d) *Stephanosphaera* (Archaeplastida [Plantae], Viridiplantae, chlorophyte), (e) *Chaetosphaeridium* (Archaeplastida [Plantae], Viridiplantae, streptophyte), (f) *Mallomonas* (stramenopiles, chrysophyte), (g) *Coscinodiscus* (stramenopiles, diatom), (h) *Synedra* (stramenopiles, diatom), (i) *Sphacelaria* (stramenopiles, brown alga), (j) *Trithigmostoma* (alveolates, ciliate), (k) *Cryptomonas* (Cryptista), (l) *Paramoeba* (Amoebozoa). Micrographs courtesy of Gerd Günther (http://www.mikroskopia.de/index.html), Sebastian Hess (Halifax; *Scherffelia*), and Ivan Fiala (Czech Republic; *Paramoeba*).

Figure 8. **Sample submission portal from the CNGB/10KP website.** Figure here shows the sample submission portal (as well as the underlying database management) on the 10KP website: https://db.cngb.org/10kp/, and this website (now is version 1.0) is still evovling. This sample submission portal is particularly prepared for land plants, samples that mostly will come from botanical gardens or botany research centers/labs worldwide. A global community effort is crucial to help supply all families and all genera. For stage 1, we anticipate more active involvement of highly-motivated and skilled labs, whereas for stage 2, we anticipate more of a

community effort to supply the majority of the remaining genera. For algae and protists, all samples will come from the public collections and channelled through CCAC where quality control will take place.

Figure 9. An overview of strategy of 10KP.

Supplementary Tables

Table 1. Statistics of the described species of embryophytes (land plants) distributed among major clades. The numbers were combined and integrated from The Open Tree of Life and The Plant List (2013)[8] especially for flowering plants, but a particular focus on the "accepted species" for the non-flowering plants due to its difficulty in classification. Some numbers are not the same or inconsistent across different databases. The aim of this table is simply to give a rough estimate of the species distribution in the major clades, and the three largest families within each clade.

Clade	Family	Genus	Species	The Three Largest Families		
**Hornworts**	5	11	130	Anthocerotaceae (161)	Dendrocerotaceae (25)	Notothyladaceae (16)
**Liverworts**	87	387	7356	Lejeuneaceae (2,270)	Jungermanniaceae(725)	Lepidoziaceae (580)
**Mosses**	111	874	13000	Pottiaceae (3,223)	Hypnaceae (2,520)	Bryaceae (2,108)
**Lycophytes**	3	18	1338	Lycopodiaceae (475)	Selaginellaceae (404)	Isoetaceae (51)
**Ferns**	48	319	10578	Dryopteridaceae (1871)	Polypodiaceae (1,601)	Pteridaceae (1,226)
**Gymnosperms**	12	88	1104	Pinaceae (255)	Zamiaceae (216)	Cupressaceae (166)
**ANA Grade**	6	20	193	Nymphaeaceae (88)	Schisandraceae (84)	Hydatellaceae (12)
**Monocots**	78	3505	76119	Orchidaceae (28,576)	Poaceae (12,397)	Cyperaceae (6,311)
**Magnoliids**	20	450	9528	Lauraceae (3,106)	Piperaceae (2,770)	Annonaceae (2,174)
**Asterids**	143	9763	135213	Asteraceae (38,700)	Rubiaceae (14,412)	Lamiaceae (8,671)
**Rosids**	154	6582	101245	Fabaceae (26,245)	Euphorbiaceae (6,904)	Rosaceae (6,626)
**Basal eudicots**	17	464	7535	Ranunculaceae (3,119)	Proteaceae (1,492)	Papaveraceae (1,062)
**Others**	NA	NA	1579			
**Embryophyta**	667	23,562	381,425			

Table 2. Technologies/platforms for DNA library construction and sequencing. The MGISEQ platform, a BGI-developed next-generation sequencing (NGS) technology based on DNA nanoballs and combinatorial Probe Anchor Synthesis (cPAS), will be combined with evolving methodologies for improving long-range contiguity, including linked-read technologies from 10X genomics or single tube Long Fragment Read (stLFR) from Complete Genomics (a division of BGI). HiC libraries may also be used to construct chromosome-level genome reference assemblies for a member of every family or for other critical species selected by the

consortium. Generally speaking, sample providers must prepare either a) minimum amount of DNA >50 ug and DNA fragments >20 kb; or b) minimum amount of DNA >5 ng and DNA fragments >50 kb or preferably >100 kb. For a), we will use the traditional hierarchical shotgun strategy by combining paired-end libraries with a series of mate-pair libraries. For b), we will use the strategy of 10X + MGISEQ. The details of these platforms and the library/sequencing strategies are summarized in Table 2.

**Platform /strategy**	**Insert size**	**Assemblers**	**Quality requirement for Tissue samples**	**Minimum requirement of DNA quantity**	**Minimum requirement of DNA fragments**	**Assembly results**
MGISEQ (hierarchical shotgun)	170/350bp	SOAPdenovo	Net weight of fresh plant tissue >=4g	2μg	>20k	construct contig
	2-5K			20μg		construct scaffold
	10k			30μg		
	20k			50μg		
	40k			60μg		
MGISEQ + 10X		Supernova2	50–75 mg dried or 100–200 mg fresh leaf tissue	2ng	>50/100k	construct scaffold
MGISEQ + LFR		to be developed	50–75 mg dried or 100–200 mg fresh leaf tissue	1ng	>50/100k	construct contig
MGISEQ + 10X/LFR + Hi-C			500 mg, living tissues/cells		>50/100k	chromosome-scale

Figure 1

Click here to download Figure Figure 1.pdf

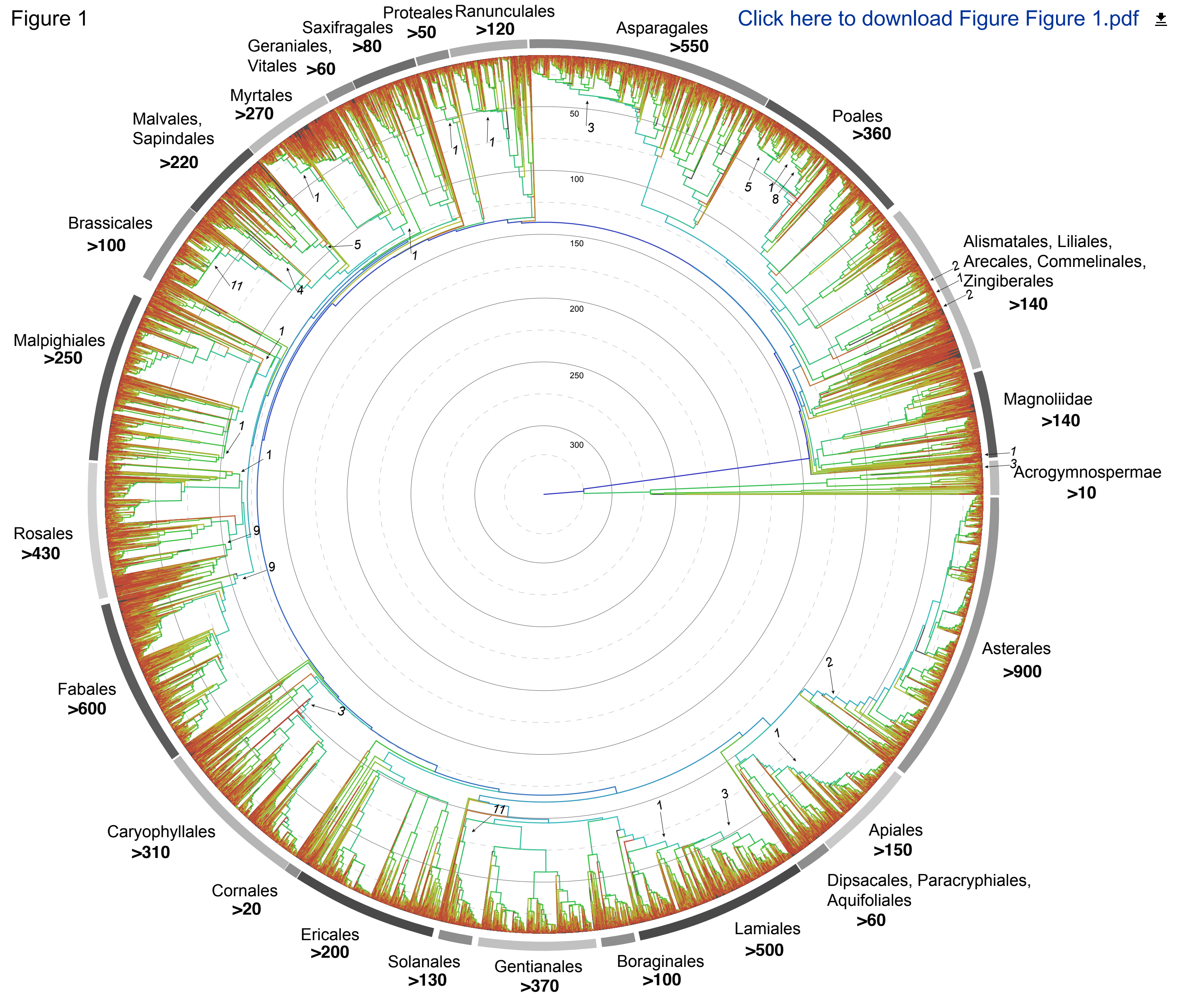

Figure 2

Click here to download Figure Figure 2.pdf

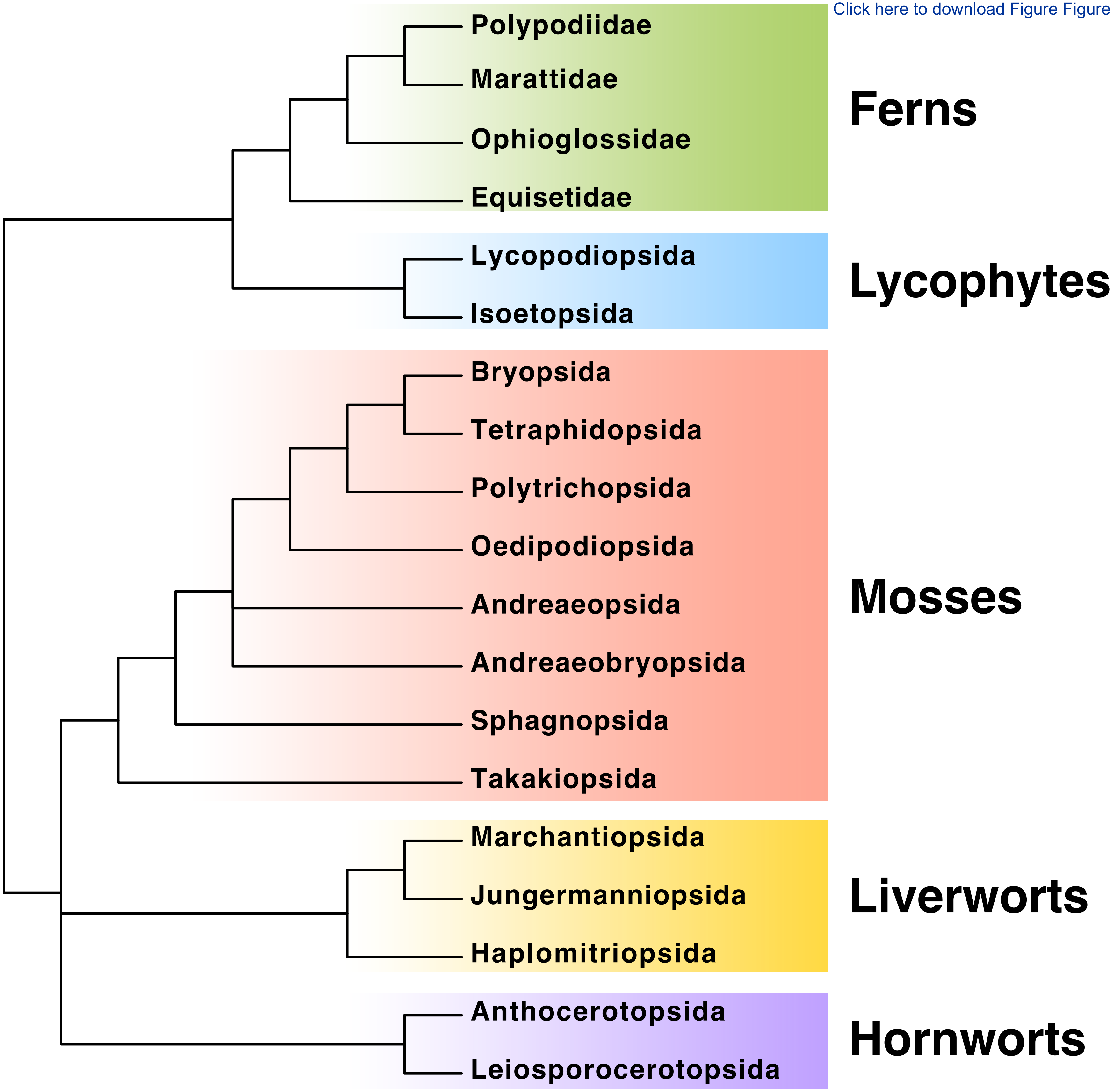

Figure 3

Click here to download Figure Figure 3.pdf

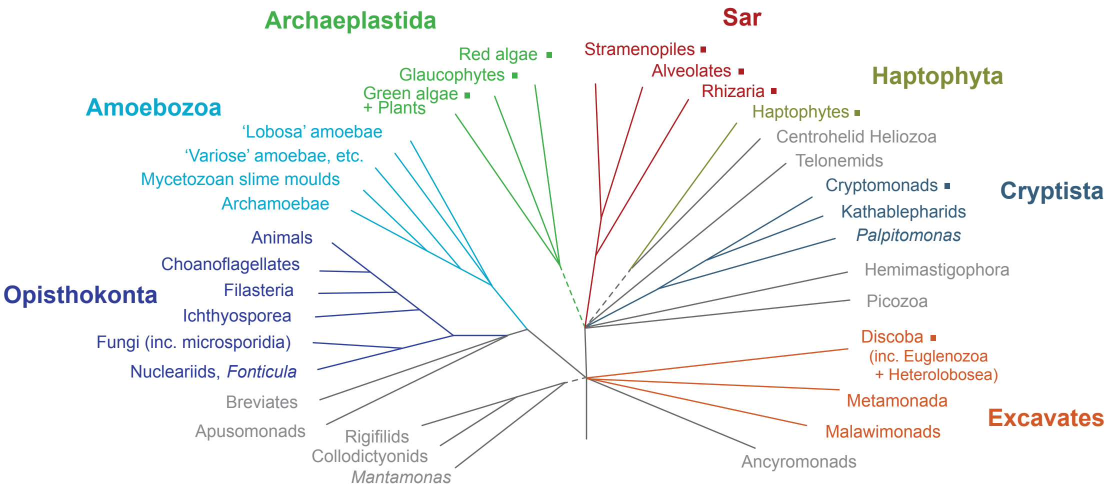

Figure 4

Click here to download Figure Figure 4.pdf

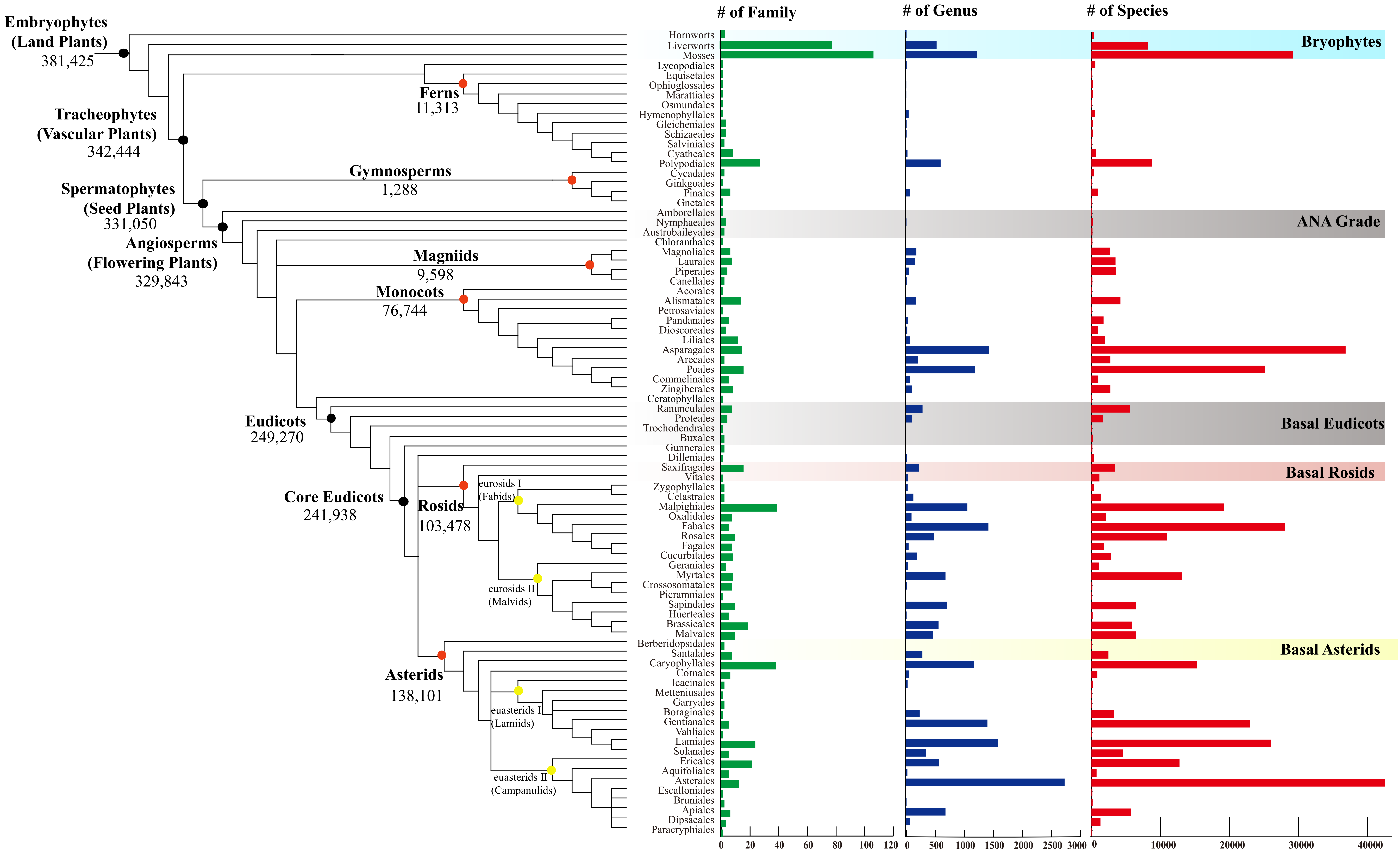

Figure 5

Click here to download Figure Figure 5.png

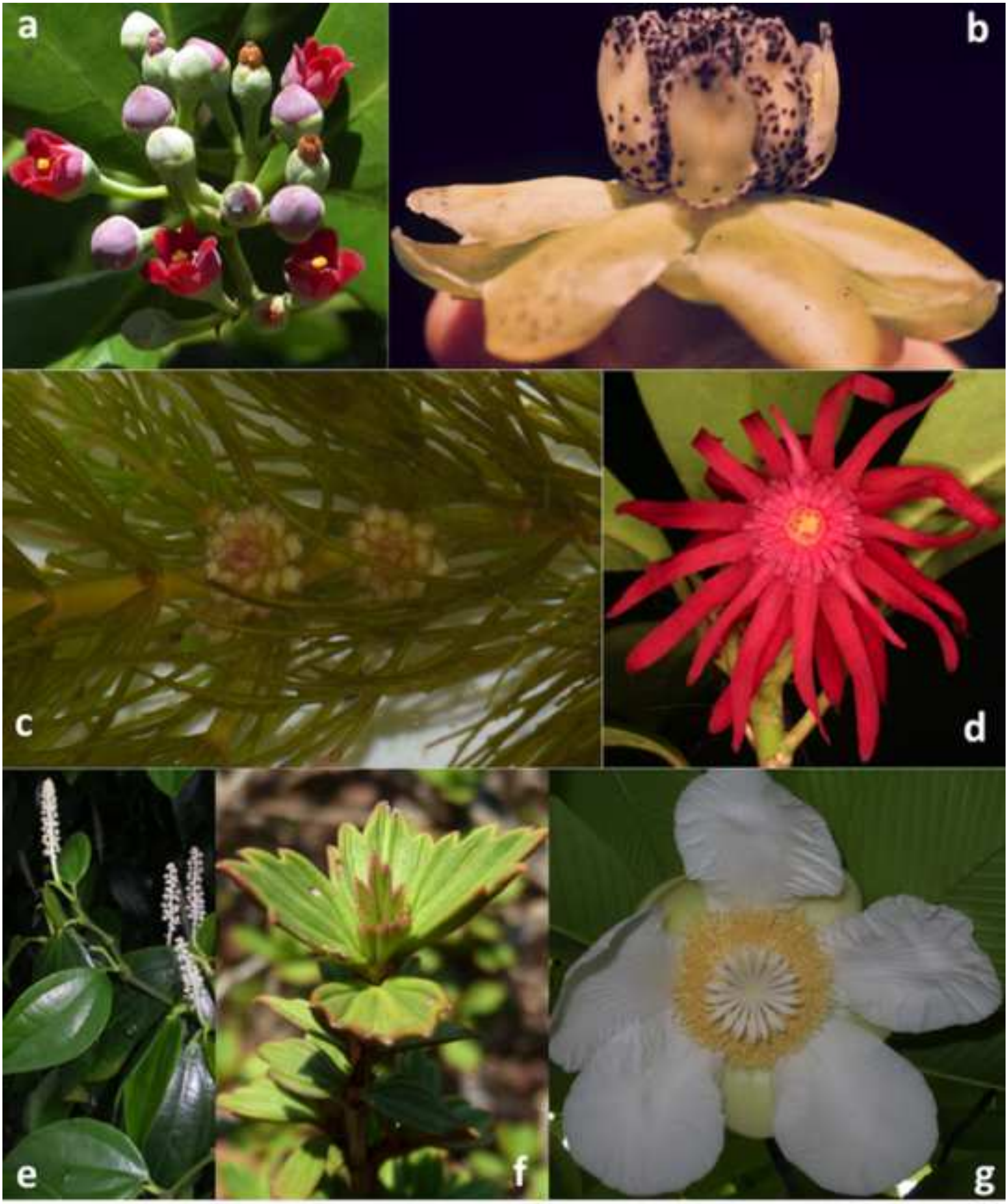

 Click here to download Figure Figure 6.png

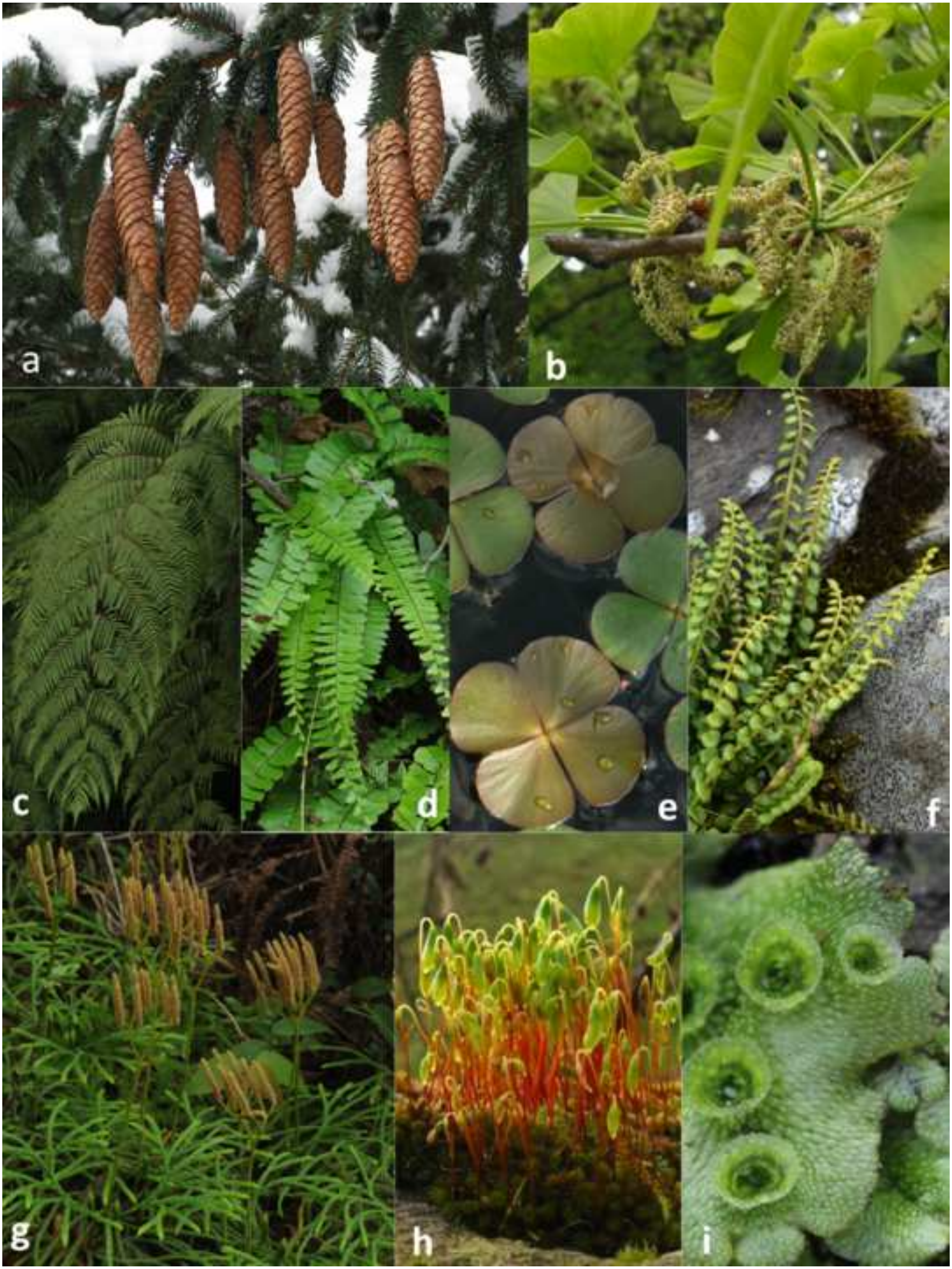

Figure 7

Click here to download Figure Figure 7.jpg

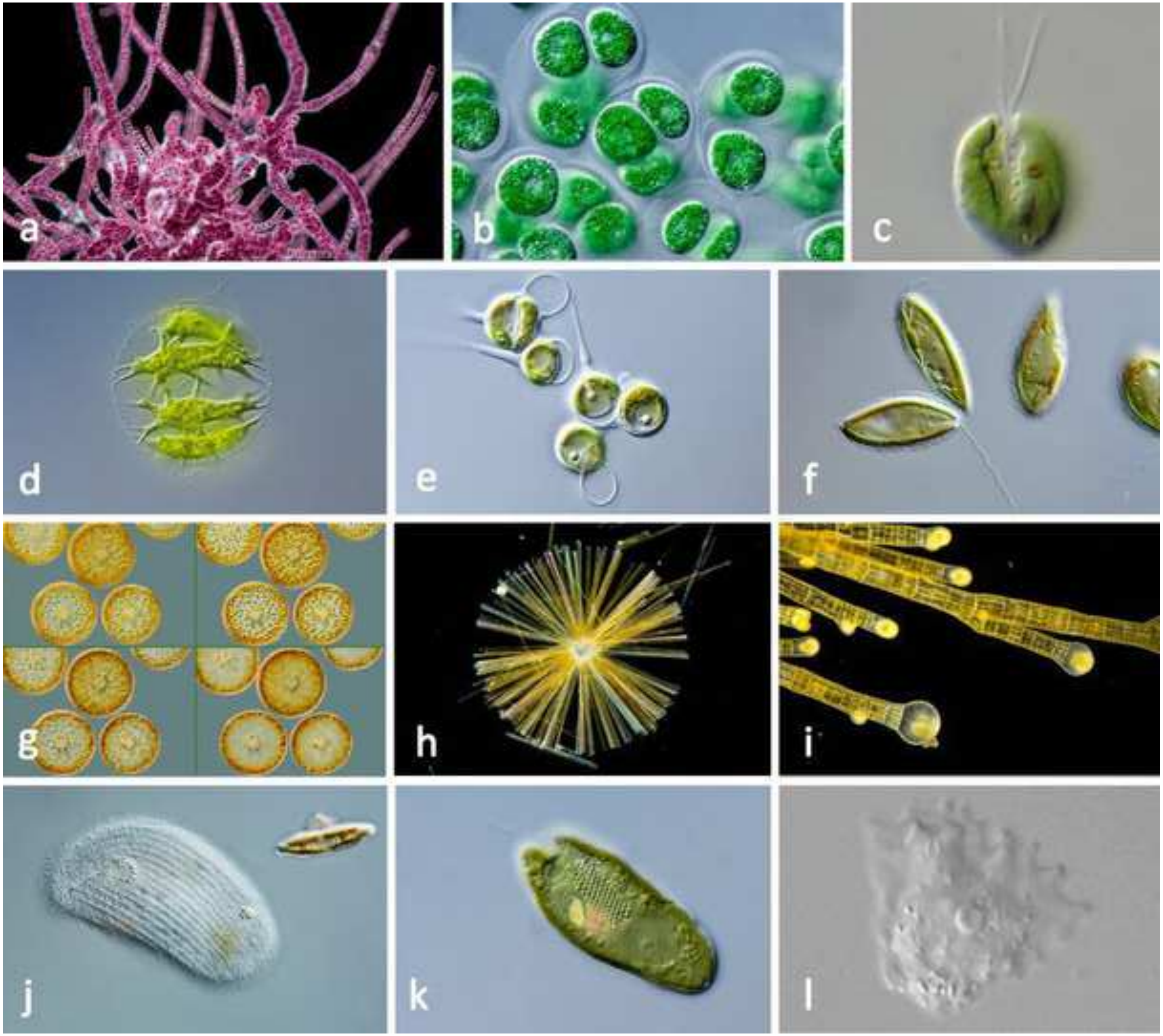

Figure 8

Click here to download Figure Figure 8.png

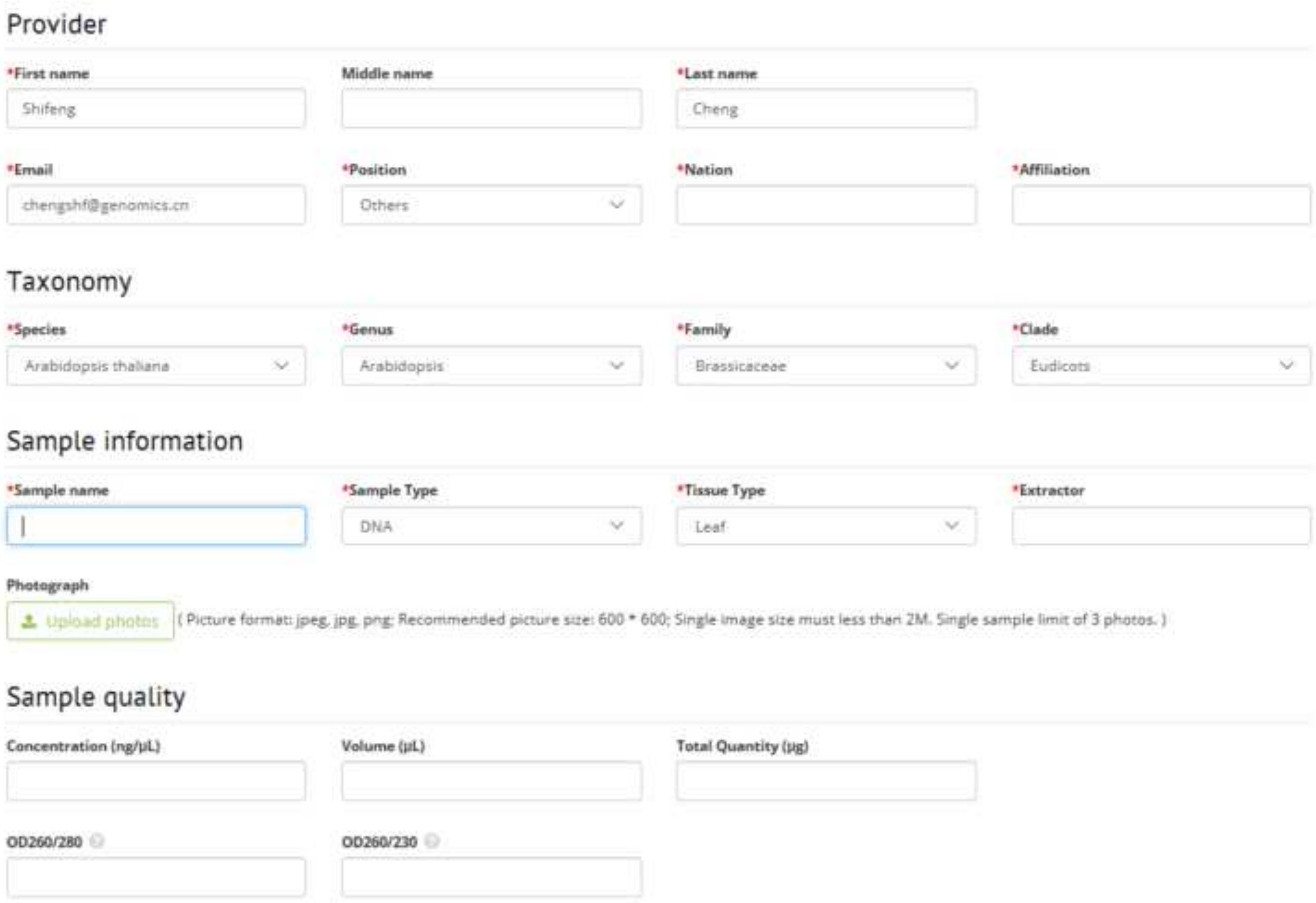

Figure 9

Click here to download Figure Figure 9.pdf

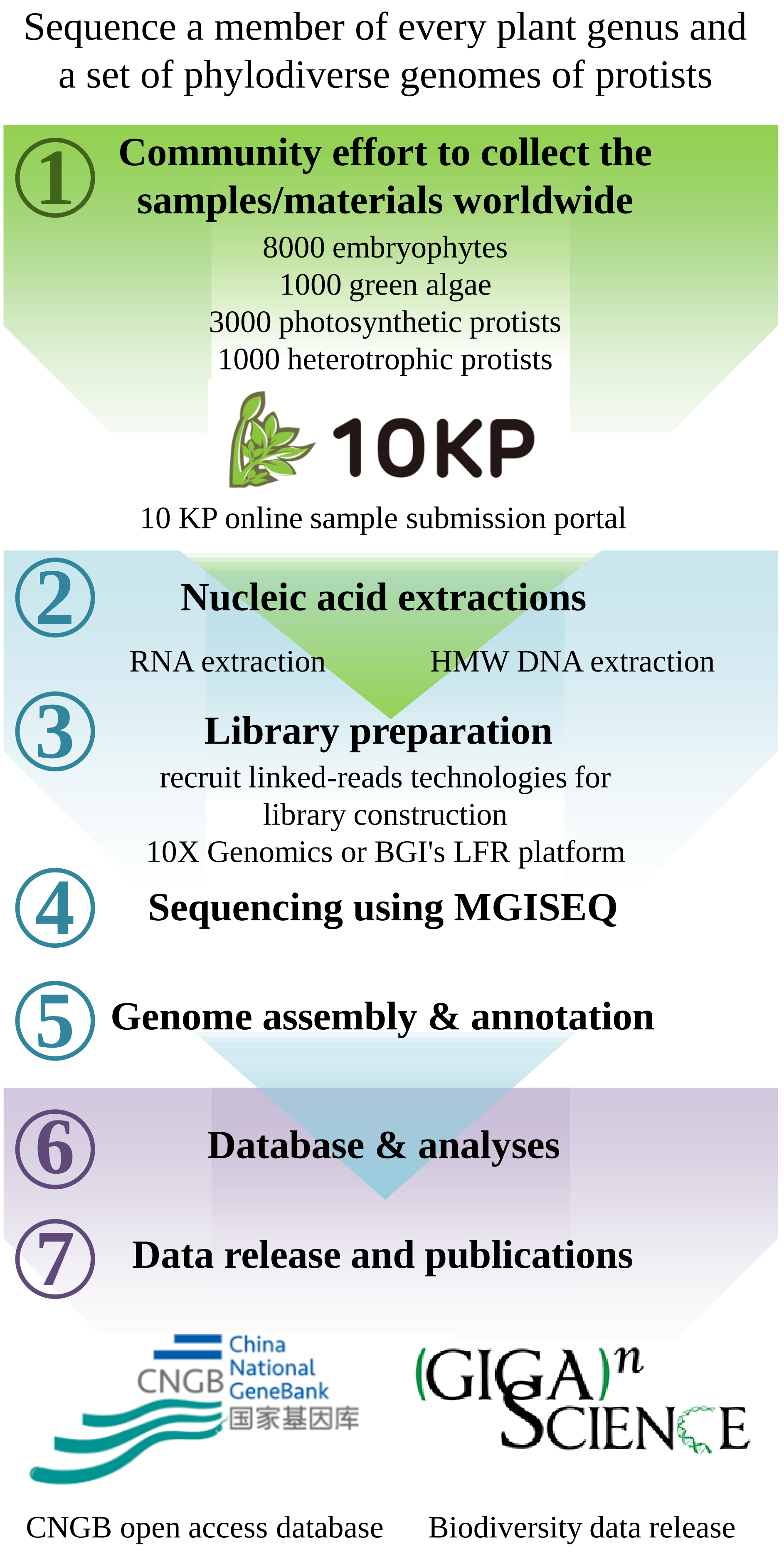

Click here to access/download
Supplementary Material
Supplementary file, proposal template.docx
